# Estimating the survival of elderly patients diagnosed with dementia in Taiwan: A longitudinal study

**DOI:** 10.1371/journal.pone.0178997

**Published:** 2018-07-25

**Authors:** Kwo-Chen Lee, Wen-Hsuan Hsu, Po-Han Chou, Jia-Jean Yiin, Chih-Hsin Muo, Yun-Ping Lin

**Affiliations:** 1 School of Nursing, China Medical University, Taichung, Taiwan; 2 Department of Nursing, China Medical University Hospital, Taichung, Taiwan; 3 Brain Disease Research Center, China Medical University Hospital, Taichung, Taiwan; 4 Department of Psychiatry, Taichung Veterans General Hospital, Taichung, Taiwan; 5 Department of Psychiatry, School of Medicine, National Yang Ming University, Taipei, Taiwan; 6 Department of Photonics, National Chiao Tung University, Hsinchu, Taiwan; 7 Department of Biological Science and Technology, National Chiao Tung University, Hsinchu, Taiwan; 8 Department of Neurosurgery, Neurological Institute, Taichung Veterans General Hospital, Taichung, Taiwan; 9 Institute of Medical Science, National Defense Medical Center, Taipei, Taiwan; 10 Management Office for Health Data, China Medical University Hospital, Taichung, Taiwan; 11 College of Medicine, China Medical University, Taichung, Taiwan; Cardiff University, UNITED KINGDOM

## Abstract

**Background:**

Dementia is characterized by prolonged progressive disability. Therefore, predicting mortality is difficult. An accurate prediction tool may be useful to ensure that end-of-life patients with dementia receive timely palliative care.

**Purpose:**

This study aims to establish a survival prediction model for elderly patients with dementia in Taiwan.

**Methods:**

Data from the 2001 to 2010 National Health Insurance Research Database in Taiwan were used to identify 37,289 patients with dementia aged ≥65 years for inclusion in this retrospective longitudinal study. Moreover, this study examined the mortality indicators for dementia among demographic characteristics, chronic physical comorbidities, and medical procedures. A Cox proportional hazards model with time-dependent covariates was used to estimate mortality risk, and risk score functions were formulated using a point system to establish a survival prediction model. The prediction model was then tested using the area under the receiver operating characteristic curve.

**Results:**

Thirteen mortality risk factors were identified: age, sex, stroke, chronic renal failure, liver cirrhosis, cancer, pressure injury, and retrospectively retrieved factors occurring in the 6 months before death, including nasogastric tube placement, supplemental oxygen supply, ≥2 hospitalization, receiving ≥1 emergency services, ≥2 occurrences of cardiopulmonary resuscitation, and receiving ≥2 endotracheal intubations. The area under the receiver operating characteristic curves for this prediction model for mortality at 6 and 12 months were 0.726 and 0.733, respectively.

**Conclusions:**

The survival prediction model demonstrated moderate accuracy for predicting mortality at 6 and 12 months before death in elderly patients with dementia. This tool may be valuable for helping health care providers and family caregivers to make end-of-life care decisions.

## Introduction

A total of 47 million people is currently estimated to have dementia worldwide, and numbers will triple by 2050 [1]. In Taiwan, >60% of nursing home residents have dementia [2]. Dementia is an incurable progressive disorder that gradually leads to the loss of cognitive function and finally to death. Studies have found that the mean survival time from diagnosis to death is 4.48 years for patients with dementia in Taiwan [3]. Severe cognitive dysfunction [4,5], functional impairment more than six months [5], taking numerous medications [5], using atypical antipsychotic medication [5], pneumonia [4,6], cardiovascular diseases [6], fractures [7], cachexia [8], dysphagia, and malnutrition [4,9] have been found to be the most common mortality factors in patients with advanced dementia. A previous study showed that the aggressive treatment of dementia may not increase survival time but does cause unnecessary suffering [4]. Estimating survival following dementia diagnosis is critical for patients and their families, because such knowledge can enable family caregivers to plan end-of-life care.

Dementia is not a direct cause of death. Therefore, accurately estimating life expectancy in patients with dementia is crucial, and the lack of an accurate prediction tool has been a major barrier to providing palliative care [10]. Various scales have been developed to define dementia indicators, but most have been found to be invalid for predicting mortality [11–13]. Furthermore, most previous studies have only used one-dimensional indicators, such as physical symptoms [14], acute illnesses [6,7] physical activity [15], or cognitive function [16] to develop mortality risk prediction tools for patients with dementia. Therefore, these tools are not based on a comprehensive or multidimensional approach to mortality prediction.

In this study, multidimensional indicators of patient demographics, comorbidities, and medical procedures were extracted from the 2001–2010 National Health Insurance Research Database (NHIRD) to identify the predictors of mortality in elderly patients with dementia. These predictors were used as a reference index for palliative care interventions in Taiwan.

## Methods

### Study sample

A retrospective cohort design with secondary analysis was adopted. This study used data from the NHIRD, which is maintained by the National Health Research Institute of Taiwan. Nearly all hospitals and clinics in Taiwan are contracted with the National Health Insurance program, which provides health care to 96% of residents through social insurance [17]. The NHIRD provides a large amount of information, including age, sex, date of mortality, dates of admission and discharge, International Classification of Diseases, Ninth Revision, Clinical Modification (ICD-9-CM) diagnostic and medical procedure codes, comorbidity, and emergency care details. Patients with a Clinical Dementia Rating (CDR) of ≥2 in the NHIRD qualified as having a catastrophic illness. In this study, the data of all patients with dementia and a CDR of ≥2 were merged with the data of “ambulatory care expenditures by visit” and “inpatient expenditures by admissions” from 2001 to 2010 and subsequent follow-up from 2001 to 2011. The inclusion criteria were (1) ≥65 years old and (2) a diagnostic code of dementia (ICD -9-CM codes: 290, 294.1, 331.0).

### Survival predictors and outcomes

The predictors in this study were selected on the basis of previous research [4–7,14,18–23] and our clinical experience. The predictors were patient demographics, such as sex and age, comorbidities, and received medical procedures. Age was stratified into 65–69, 70–74, 75–79, 80–84, and ≥85 years. Comorbidities were hypertension, coronary artery disease (CAD), chronic heart failure (CHF), diabetes, cancer, stroke, chronic renal failure, hyperlipidemia, chronic obstructive pulmonary disease (COPD), liver cirrhosis, and pressure injury. Hospitalization parameters were ≥2 ward care and receiving ≥1 emergency services. Medical procedures were nasogastric (NG) tube placement, supplemental oxygen supply, receiving ≥2 endotracheal intubations (ETs), and ≥2 occurrences of cardiopulmonary resuscitation (CPR) in the 6 months before patient death. Patients who had received only 1 ET or 1 CPR (indicating that the patients possibly died at that time) were excluded. Patients were followed up from the diagnosis of dementia with a CDR of ≥2 until death. Patients who did not die during the study period were followed up until the date of withdrawal from the National Health Insurance program or to the end of 2010, whichever came first.

### Statistical analysis

Descriptive analysis of all variables was performed using SAS 9.3 software (SAS Institute, Cary NC). Subsequently, all participants were randomized at a 2:1 ratio into a derivation group and a validation group (standardized mean difference range: 0.011–0.024 standard deviations). Thereafter, univariate and multivariate analyses were performed to identify significant predictors. The mortality risk for each variable was estimated using the incidence density from the Cox proportional hazards model, and significant variables and risk scores >0 were included in the adjustments used in the predictive model. The model was adjusted for age, sex, cancer, stroke, chronic renal failure, liver cirrhosis, pressure injury, hospitalizations, receiving emergency services, NG tube placement, oxygen supply, receiving CPRs, and receiving ETs. Comorbidities were classified as time-dependent variables because they changed during follow-up. The age categories used in this study were the same as those used in the Framingham study [24], with 5-year intervals for each age category, except for the last one, which was open-ended. The inclusion of these variables in the multivariable model-building procedure was guided by the relative strength of statistical significance, collinearity, and expert opinions. The midpoints of each age category were used as the reference values to calculate the risk score. Finally, in the validation group, the area under the receiver operating characteristic curve (AUROC) was used for curve analysis and predicting the level of accuracy [25].

## Results

### Study sample

A total of 37,289 elderly patients with dementia (40.8% women) were included in this study, with an average age of 78.98 ± 6.71years. The median and maximum survival times from dementia diagnosis to death from 2001 to 2011 were 3.28 and 5.59 years, respectively.

The prevalence of comorbidities, such as CAD, CHF, diabetes, stroke, chronic renal failure, hyperlipidemia, COPD, cancer, liver cirrhosis, pressure injury was significantly higher in the deceased group than in the alive group. The proportion of patients who were hospitalized or received medical procedures was significantly higher in the deceased group than in the alive group. Moreover, in the deceased group, 46% of patients had ≥2 ward care and 65% had received ≥1 emergency services. Approximately 70% of patients who received a diagnosis of dementia 6 months before their death had undergone NG tube placement and received supplemental oxygen supply; moreover, 31.8% of deceased patients had received ≥2 ETs, and 49.9% had received ≥2 CPRs (Table 1).

**Table 1:** Characteristics of the derivation and validation groups of elderly patients diagnosed with dementia.

Characteristics	Derivation subgroup (n = 24859)				P value	Validation subgroup (n = 12430)				P value
	Alive (n = 11210)		Deceased (n = 13649)			Alive (n = 5537)		Deceased (n = 6893)		
	n	%	n	%		n	%	n	%	
**Sex**					<0.0001					<0.0001
Male	7358	(49.8)	7412	(50.2)		3637	(49.6)	3690	(50.4)	
Female	3852	(38.2)	6237	(61.8)		1900	(37.2)	3203	(62.8)	
**Age**, years					<0.0001					<0.0001
65–69	1581	(61.2)	1001	(38.8)		718	(59.7)	484	(40.3)	
70–74	2488	(53.3)	2180	(46.7)		1343	(55.0)	1101	(45.0)	
75–79	3113	(47.3)	3474	(52.7)		1532	(46.8)	1745	(53.2)	
80–84	2522	(40.4)	3720	(59.6)		1249	(39.6)	1906	(60.4)	
≥85	1506	(31.5)	3274	(68.5)		695	(29.6)	1657	(70.4)	
Mean (SD)^†^	77.6	(6.52)	80.1	(6.67)	<0.0001	77.5	(6.4)	80.2	(6.7)	<0.0001
**Comorbidity**										
Hypertension	9103	(81.2)	11187	(82.0)	0.12	4480	(80.9)	5661	(82.1)	0.08
CAD	5393	(48.1)	6869	(50.3)	0.0005	2689	(48.6)	3466	(50.3)	0.06
CHF	2374	(21.2)	3819	(28.0)	<0.0001	1170	(21.1)	1969	(28.6)	<0.0001
Hyperlipidemia	5859	(52.3)	5092	(37.3)	<0.0001	2895	(52.3)	2513	(36.5)	<0.0001
Diabetes	3789	(33.8)	4945	(36.2)	<0.0001	1894	(34.2)	2568	(37.3)	0.0004
Cancer	842	(7.51)	1752	(12.8)	<0.0001	429	(7.8)	851	(12.4)	<0.0001
Stroke	4244	(37.9)	7145	(52.4)	<0.0001	2105	(38.0)	3606	(52.3)	<0.0001
Chronic Renal failure	1505	(13.4)	2815	(20.6)	<0.0001	772	(13.9)	1406	(20.4)	<0.0001
COPD	4722	(42.1)	7384	(54.1)	<0.0001	2336	(42.2)	3630	(52.7)	<0.0001
Liver Cirrhosis	214	(1.91)	414	(3.03)	<0.0001	105	(1.9)	219	(3.18)	<0.0001
Pressure injury	4722	(42.1)	7384	(54.1)	<0.0001	2336	(42.2)	3630	(52.7)	<0.0001
**Hospitalization**										
Ward care ≥ 2 times	115	(10.0)	6388	(46.8)	<0.0001	544	(9.8)	3119	(45.3)	<0.0001
Emergency services ≥ 1 times	3165	(28.2)	8863	(64.9)	<0.0001	1565	(28.3)	4412	(64.0)	<0.0001
**Medical procedure**										
NG tube placement	2858	(25.5)	10517	(77.1)	<0.0001	1453	(26.2)	5311	(77.1)	<0.0001
Oxygen supply	2104	(18.8)	9519	(69.7)	<0.0001	1052	(19.0)	4825	(70.0)	<0.0001
ET ≥ 2 times	323	(2.9)	4338	(31.8)	<0.0001	135	(2.4)	2140	(31.1)	<0.0001
CPR ≥ 2 times	609	(5.4)	6810	(49.9)	<0.0001	277	(5.0)	3368	(48.9)	<0.0001

CAD, coronary artery disease; CHF, chronic heart failure; COPD, chronic obstructive pulmonary disease

NG, nasogastric; ET, endotracheal intubation; CPR, cardiopulmonary resuscitation

Chi-square test and ^†^ t-test

#### Predictors 6 months before death

The univariate and multivariate analyses revealed the significant mortality factors for elderly patient with dementia in the derivation group in Table 2. Hypertension, CAD, and hyperlipidemia were found to be significant protective factors against death in the Cox proportional hazard model with time-dependent covariates. Multivariate analysis demonstrated that age, sex, CHF, diabetes, cancer, stroke, chronic renal failure, COPD, liver cirrhosis, pressure injury, ≥2 ward care, ≥1 emergency services, NG tube placement, oxygen supply, and receiving ≥2 ETs or ≥2 CPRs in the 180 days before death were the mortality factors that significantly affected life expectancy in elderly patients with dementia. The multivariable model revealed significant differences in 17 variables (p <0.0001). Because the chi-square criterion score for CAD, CHF, hyperlipidemia, and diabetes was 0, we excluded these variables from the final prediction model. The thirteen variables that most accurately predicted survival were selected (Table 3): sex, age, cancer, stroke, chronic renal failure, liver cirrhosis, pressure injury, ≥2 ward care, ≥1 emergency services, NG tube placement, oxygen supply, receiving ≥2 ETs or ≥2 CPRs in the 6 months before death.

**Table 2:** Characteristics of elderly patients diagnosed with dementia and their unadjusted associations with mortality in the derivation group determined by using Cox proportional hazards regression with time-dependent covariates.

	Univariate analysis		Multivariate analysis	
Characteristics	Crude HR (95% CI)	P value	Adjusted HR (95% CI)	P value
**Age**, years				
65–69	1.00		1.00	
70–74	1.29 (1.19–1.39)	<0.0001	1.13 (1.05–1.22)	0.0011
75–79	1.62 (1.51–1.74)	<0.0001	1.34 (1.24–1.43)	<0.0001
80–84	2.12 (1.98–2.28)	<0.0001	1.72 (1.60–1.84)	<0.0001
85+	3.12 (2.90–3.35)	<0.0001	2.45 (2.28–2.64)	<0.0001
**Sex**				
Female	1.00		1.00	
Male	1.41 (1.36–1.45)	<0.0001	1.20 (1.16–1.25)	<0.0001
**Comorbidity**				
Hypertension	1.14 (1.09–1.19)	<0.0001	0.93 (0.89–0.97)	0.0018
CAD	1.12 (1.08–1.16)	<0.0001	0.92 (0.89–0.96)	<0.0001
CHF	1.46 (1.41–1.52)	<0.0001	1.10 (1.05–1.14)	<0.0001
Hyperlipidemia	0.82 (0.79–0.85)	<0.0001	0.92 (0.89–0.96)	<0.0001
Diabetes	1.17 (1.14–1.22)	<0.0001	1.14 (1.09–1.18)	<0.0001
Cancer	1.75 (1.65–1.85)	<0.0001	1.71 (1.61–1.81)	<0.0001
Stroke	1.69 (1.63–1.74)	<0.0001	1.37 (1.32–1.42)	<0.0001
Chronic Renal failure	1.77 (1.69–1.85)	<0.0001	1.43 (1.36–1.50)	<0.0001
COPD	1.77 (1.69–1.85)	<0.0001	1.06 (1.02–1.10)	0.0015
Liver cirrhosis	1.68 (1.51–1.86)	<0.0001	1.33 (1.20–1.48)	<0.0001
Pressure injury	2.02 (1.91–2.13)	<0.0001	1.42 (1.34–1.50)	<0.0001
**Hospitalization**				
Ward care ≥ 2 times	2.83 (2.74–2.93)	<0.0001	1.20 (1.15–1.25)	<0.0001
Emergency services ≥ 1 times	2.54 (2.45–2.63)	<0.0001	1.24 (1.20–1.29)	<0.0001
**Medical procedure**				
NG tube placement	3.72 (3.58–3.87)	<0.0001	1.52 (1.44–1.60)	<0.0001
Oxygen supply	3.61 (3.48–3.75)	<0.0001	1.54 (1.46–1.61)	<0.0001
ET ≥ 2 times	3.96 (3.83–4.10)	<0.0001	1.88 (1.80–1.96)	<0.0001
CPR ≥ 2 times	3.27 (3.15–3.39)	<0.0001	1.51 (1.45–1.58)	<0.0001

CAD, Coronary artery disease; CHF, chronic heart failure; COPD, chronic obstructive pulmonary disease; NG, nasogastric; ET, endotracheal intubation; CPR, cardiopulmonary resuscitation.

**Table 3:** Predicted risk scores for mortality in elder dementia patients using Cox proportional hazard regression models in derivation group.

Characteristics	Adjusted HR (95% CI)	*β*	Points in risk score
**Sex**			
Male	1.21 (1.17–1.26)	0.19300	1
Female	1.00 (Ref.)	0	0
**Age**, years			
65–69	1.00 (Ref.)	0	0
70–74	1.13 (1.05–1.22)	0.12456	1
75–79	1.33 (1.24–1.43)	0.28493	2
80–84	1.71 (1.59–1.83)	0.53616	3
≥85+	2.45 (2.28–2.63)	0.89657	4
**Comorbidity**			
Cancer	1.70 (1.61–1.80)	0.53151	2
Stroke	1.37 (1.32–1.41)	0.31198	1
Renal failure	1.43 (1.37–1.50)	0.35911	1
Liver cirrhosis	1.36 (1.22–1.51)	0.30543	1
Pressure injury	1.45 (1.37–1.53)	0.36965	2
**Hospitalization**			
Ward care ≥ 2 times	1.21 (1.16–1.26)	0.19020	1
Emergency services ≥ 1 times	1.24 (1.19–1.29)	0.21432	1
**Medical procedure**			
NG tube placement	1.53 (1.46–1.61)	0.42955	2
Oxygen supply	1.54 (1.47–1.62)	0.43172	2
ET ≥ 2 times	1.88 (1.80–1.97)	0.63292	3
CPR ≥ 2 times	1.51 (1.45–1.58)	0.41409	2

NG, nasogastric; ET, endotracheal intubation; CPR, cardiopulmonary resuscitation.

#### Risk score derivation and accuracy

The midpoints for each 5-year age category (same as the categories used in the Framingham study risk score) were used as the ratios to calculate the score for each variable. The score range for the mortality risk prediction model was 0–23, with higher scores indicating a higher mortality risk (Table 4). Patients aged ≥85 years had the highest scores, followed by those aged 80–84 years and those who had received ≥ 2 ETs in the 6 months before death (Table 3). Table 4 shows that approximately 50% of the patients with scores between 16 and 17 died within 6 months, whereas those with scores between 14 and 15 died within 12 months.

**Table 4:** Number and percentage of patients with each possible risk score and 6- and 12-month probabilities of death for each score calculated from the derivation group.

Cumulative Risk score	Number of subjects with each score		Estimated probabilities of death within	
	N	%	6 months	12 months
**0**	388	1.56	0.0112	0.0223
**1**	1459	5.87	0.0142	0.0283
**2**	3254	13.09	0.0181	0.0360
**3**	5428	21.84	0.0231	0.0456
**4**	7623	30.66	0.0293	0.0578
**5**	9329	37.53	0.0372	0.0732
**6**	10765	43.30	0.0472	0.0924
**7**	11998	48.26	0.0598	0.1163
**8**	13503	54.32	0.0757	0.1458
**9**	15205	61.16	0.0955	0.1821
**10**	17012	68.43	0.1202	0.2262
**11**	18830	75.75	0.1507	0.2790
**12**	20466	82.33	0.1880	0.3411
**13**	21923	88.19	0.2333	0.4126
**14**	23116	92.99	0.2874	0.4927
**15**	23984	96.48	0.3509	0.5792
**16**	24482	98.48	0.4238	0.6684
**17**	24742	99.53	0.5050	0.7554
**18**	24832	99.89	0.5921	0.8340
**19**	24854	99.98	0.6814	0.8988
**20**	24857	99.99	0.7675	0.9461
**21**	24858	100.00	0.8444	0.9759
**22**	24859	100.00	0.9068	0.9914
**23**	24859	100.00	0.9515	0.9977

**Fig 1:**
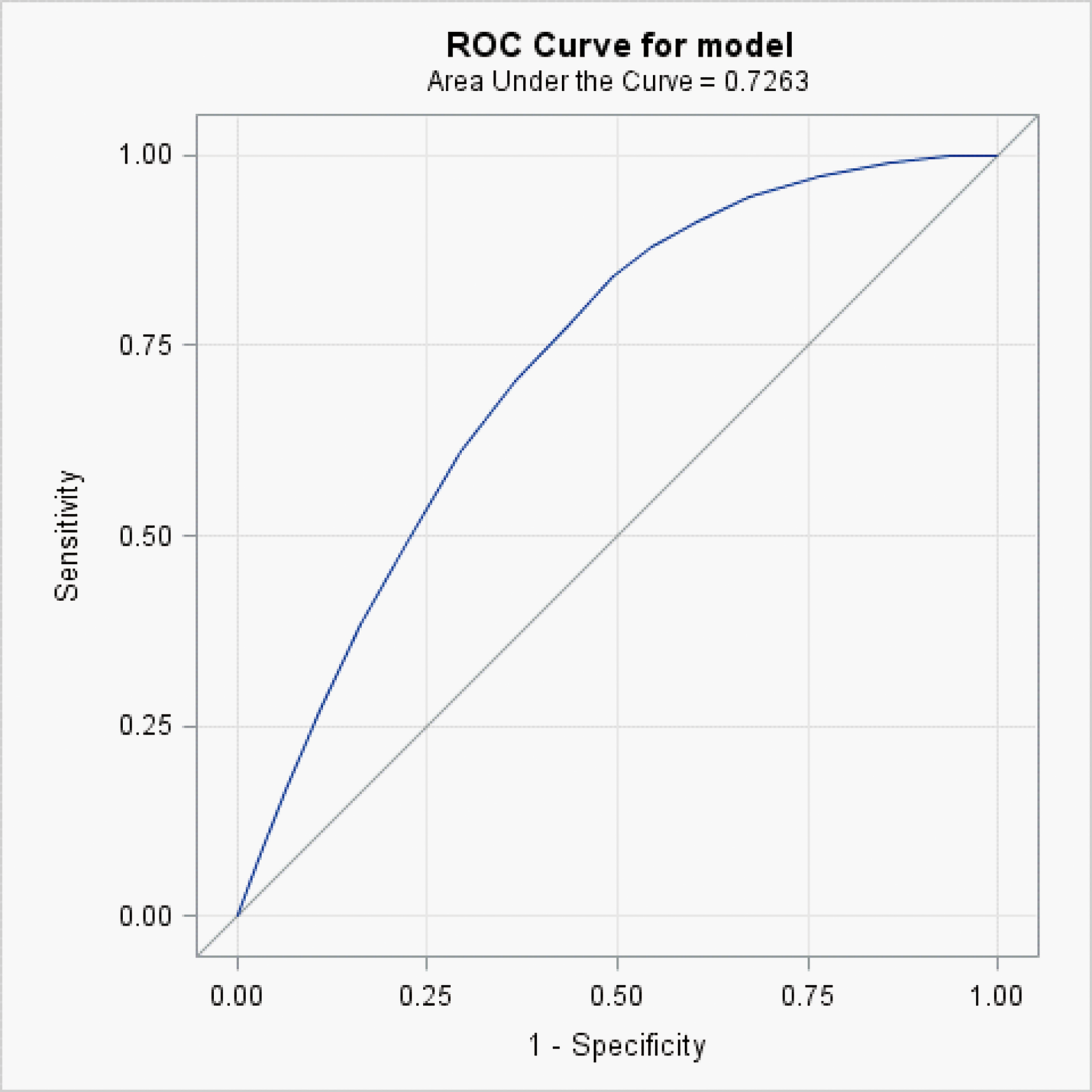
Area under the receiver operating characteristic curve (AUROC) for prediction of 6-month survival in elderly patients diagnosed with dementia.

**Fig 2:**
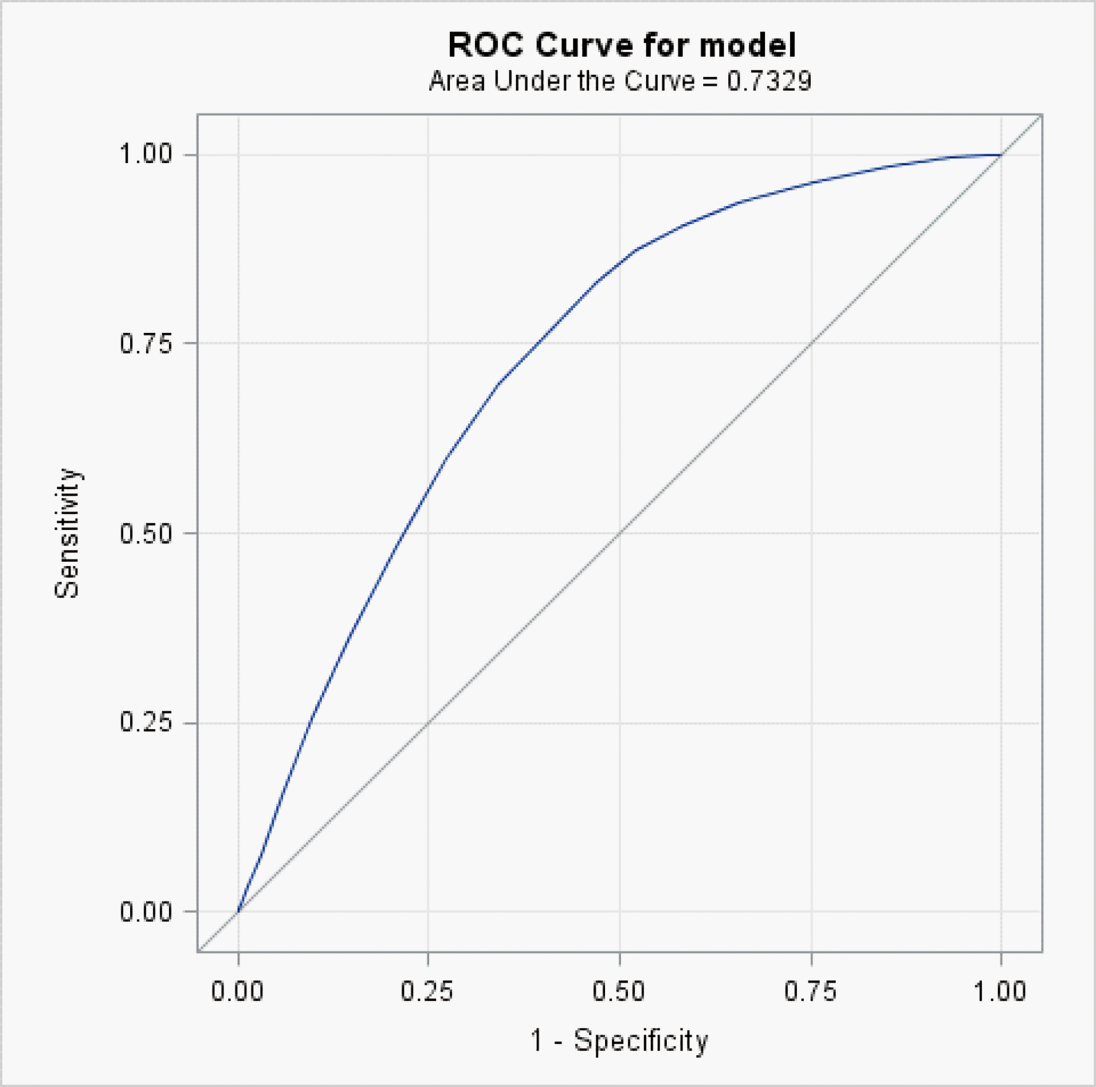
Area under the receiver operating characteristic curve (AUROC) for prediction of 12-month survival in elderly patients diagnosed with dementia.

Based on the mortality risk prediction model verified using the validation group, the AUROCs at 6 and 12 months were 0.726 (Fig 1) and 0.733 (Fig 2), respectively.

## Discussion

This study used data from the 2000–2010 NHIRD, and the multidimensional indicators of patient demographics, comorbidities, hospitalization, and medical procedures were used to predict the risk of mortality in elderly patients diagnosed with dementia. Similar to previous studies, our study suggested that sex [5,20–22], age [5,18,21], CHF [22,23,26], diabetes [6,21,26], cancer [18,20,22], stroke [6], chronic renal failure [26], and COPD [26] were significant risk factors for mortality in the Cox proportional hazards model. Similar to prior research, we found that the risk of mortality in patients with dementia increased with age [18,21], and male patients with dementia had a higher mortality rate [5,20–23]. Unlike previous studies [14,27], our study found that liver cirrhosis was a significant mortality predictor in elderly patients with dementia. Liver cirrhosis is one of the 10 leading causes of death in Taiwan [28], and it has long been regarded as the most important premalignant lesion of hepatocellular carcinoma [29].

The present study showed that among the comorbidities studies, cancer was associated with the highest mortality rate. Cancer has been the top cause of death in Taiwan for 33 years [28], and most physicians and family members choose palliative care for elderly cancer patients diagnosed with dementia because of the discomfort caused by cancer treatments. Cognitive impairment is common in elderly adults approaching the end of life in Eastern and Western countries [30].

In our study, hyperlipidemia was found to be a protective factor against death in the multivariate Cox proportional hazards model. Although high lipid levels are usually associated with the risk of cardiovascular disease and death, some observational studies have demonstrated the protective effect of high cholesterol levels and hypertension on life expectancy in the elderly patients [31].

In the present study, univariate analysis showed that hypertension was a significant risk factor for mortality. However, after controlling for diabetes and heart associated disease in the multivariate analysis, hypertension became a protective factor against mortality. The same is true for CAD. After controlling for CHF, hospitalization, and medical procedures in multivariate analysis, CAD became a protective factor against mortality. In a review and meta-analysis, van de Vorst et al. [31] indicated that patients with dementia and coronary heart disease (CHD) had a marginally higher risk of mortality than those without CHD. Nevertheless, in our study, hypertension and CAD were excluded from the final prediction model because their risk scores were zero.

Pressure injury was one of the mortality predictors included in this study. Previous studies have highlighted that higher level of functional dependency increases the risk of mortality [22], and that the incidence of pressure injury is related to physical activity impairment, with significantly lower survival rates for patients with bedsores than for those without bedsores [32]. In addition, infection resulting from pressure injury has been found to increase the risk of mortality [33].

Patients with dementia who had received ≥2 ward care or ≥1 emergency services died within 6 months. This finding is consistent with those of previous studies [7,34]. Moreover, our study showed that receiving ≥1 emergency services was a significant predictor of mortality.

Our study also found that 77% of patients with dementia who had undergone NG tube placement died within 6 months. Therefore, NG tube placement is an important risk factor for mortality. Empirical data have shown that the incidence rates of aspiration or pneumonia could not be lowered in advanced patients with dementia and NG tube placement, and their nutrition indicators could not be increased [35]. We also found that NG tube placement may increase the risk of mortality because it may cause comorbidities such as luminal perforation, aspiration-induced lung injury, or inadvertent intracranial placement of a NG tube [36].

Supplemental oxygen supply was also a significant mortality risk factor in patients with dementia, similar to the results from Mitchell et al. (2004, 2010) [22,26].This may be because the high frequency oxygen treatment is linked to decreased oxygen saturation. Previous studies have demonstrated that increased respiration rates and low oxygen saturation are indicators of mortality risk for elderly people [9,37].

This study is the first to show that ≥2 occurrences of ET or CPR are significant mortality factors in patients with dementia in the 6 months before their death. Further investigation is needed to determine the reason and elucidate the mechanism.

In addition to physical symptoms [14], acute illnesses [6,7], physical function [5,15], and cognitive function [5,16], as examined in previous studies, multidimensional assessments of patient demographics, comorbidities, and medical procedures were performed in the present study to construct a more comprehensive mortality risk prediction model for elderly patients diagnosed with dementia. Furthermore, the 13 indicators used in our prediction model are simple and clearly defined. Thus, clinicians can conduct a rapid assessment using this prediction model, assisting family members in making informed medical decisions. The AUROCs for this prediction model for mortality at 6 and 12 months before death were both similar [22,26] or superior to [15] those previously developed prediction tools for the mortality risk of patients with dementia.

Nevertheless, this study had some limitations. First, the study uses data that is now 8–17 years old and so may no longer reflect current management (given changing attitudes to dementia and growth in palliative approach). Second, the study did not distinguish between different dementia types (degenerative and vascular dementia survival may differ). Third, variables such as laboratory values, symptom severity, mental state, and physical capacity were not recorded in the database; thus, they were not included in this study. Fourth, 4% of medical institutions have not been contracted with the National Health Insurance program. Therefore, omissions may have occurred, because a small number of patients with dementia may have not been diagnosed or treated. Finally, the findings may not generalise to other countries.

## Conclusions

Data from the 2001–2010 NHIRD and multidimensional indicators were used in this study to retrospectively predict the risk of mortality in patients with dementia. Sex, age, cancer, stroke, chronic renal failure, liver cirrhosis, pressure injury, ≥2 ward care, ≥1emergency services, NG tube placement, supplemental oxygen supply, and receiving ≥2 ETs or ≥2 CPRs were significant risk factors for mortality prediction in patients with dementia. The prediction of this mortality risk model was moderate accuracy at both 6 and 12 months. The sample size for this prediction model was large, the predictors were simple, and patient information was easily obtained. Therefore, the results of this study should enable health care providers and family caregivers to make appropriate treatment decisions.
